# Factors that influence treatment decision-making in elderly DLBCL patients: a case vignette study

**DOI:** 10.1007/s00277-015-2358-3

**Published:** 2015-04-14

**Authors:** M. W. M. van der Poel, W. J. Mulder, G. J. Ossenkoppele, E. Maartense, M. Hoogendoorn, P. Wijermans, H. C. Schouten

**Affiliations:** 1 Department of Internal Medicine, Section of Hematology, Maastricht University Medical Centre, PO Box 5800, 6202 AZ Maastricht, The Netherlands; 2 Department of Internal Medicine, Maastricht University Medical Centre, Maastricht, The Netherlands; 3 Department of Hematology, VU University Medical Centre, Amsterdam, The Netherlands; 4 Department of Internal Medicine, Reinier de Graaf Hospital, Delft, The Netherlands; 5 Department of Hematology, Medical Centre Leeuwarden, Leeuwarden, The Netherlands; 6 Department of Hematology, Haga Hospital, The Hague, The Netherlands

**Keywords:** Diffuse large B-cell lymphoma, Elderly, Treatment decision-making, Comorbidity

## Abstract

Elderly patients with diffuse large B-cell lymphoma (DLBCL) are frequently not treated with standard immunochemotherapy, and this influences survival negatively. The purpose of this study was to gain more insight into treatment decision-making by hematologists. Case vignettes concerning patients with DLBCL were presented to hematologists in the Netherlands. Patient characteristics (age, comorbidity) differed per case. Respondents were asked in each case if they would treat the patient with curative intent by means of full-dose chemotherapy or chemotherapy with dose reduction or if they would not treat the patient with curative intent. The vast majority of respondents would treat an elderly patient diagnosed with DLBCL without a relevant medical history with full-dose chemotherapy irrespective of age. In the presence of comorbidity, lack of social support, cognitive disorders, and untreated depression dose reductions in advance are frequently applied or patients are not treated with curative intent. This is most pronounced for patients aged older than 80 years. Respondents working in a university hospital more frequently refrain form full-dose chemotherapy with curative intent compared to respondents working in tertiary medical teaching hospitals or general hospitals. Patients without a relevant medical history are generally treated with curative intent irrespective of age. Cognitive disorders, comorbidity, and depression reduce the change of being treated with curative intent. This is most prominent in the eldest patient category.

## Introduction

The incidence of non-Hodgkin’s lymphoma (NHL) increases with age, and currently, the mean age at diagnosis is 66 years [1]. Diffuse large B-cell lymphoma (DLBCL) is the most common type of aggressive NHL. Due to aging of the population, clinicians will increasingly be confronted with elderly patients diagnosed with DLBCL.

Standard treatment for patients with DLBCL consists of rituximab, cyclophosphamide, doxorubicin, vincristine, and prednisolone (R-CHOP). Not only in younger but also in elderly patients, this treatment schedule improves complete remission rates and survival [2–9]. However, in daily practice, elderly patients frequently do not receive standard immunochemotherapy treatment [4, 7, 9–12]. Reasons for suboptimal treatment are comorbidity and poor performance status, but also high age alone is adduced as an argument to refrain from standard treatment [4, 7, 10–12].

Little is known about the influence of patient characteristics on treatment decision-making by clinicians. In a recent survey among hematologists, we observed that comorbidities, cognitive disorders, and functional status are frequently taken into consideration in treatment decision-making [13]. In the second part of this survey, case vignettes of DLBCL patients with varying age and extent of comorbidity were presented to the respondents. By means of case vignettes, more information is gathered about decision-making in the daily clinical practice. Here, we present the results of the second part of this survey.

## Methods

### Data collection

Hematologists were invited to complete the online questionnaire “Treatment of the elderly with a hematologic malignancy” on behalf of the Dutch-Belgian Cooperative Trial Group for Hemato-Oncology (HOVON). HOVON is a foundation that focuses on improving and promoting treatment methods for adult patients with malignant hematologic disorders [14]. Hematologists were invited to participate through e-mail in November 2011. Non-respondents were sent a reminder e-mail within 2 months.

### Study measures

The questionnaire consisted of two parts. The first part contained questions about the importance of various factors that play a role in the decision-making of hematologists regarding treatment with curative intent in elderly patients. The results of this part were described previously [13].

In the second part of the questionnaire, case vignettes were presented to the respondents. The survey contained a total of 11 case vignettes. All cases concerned patients with DLBCL; however, the patient characteristics differed per case. In each case, a distinction was made in three age categories: patients older than 60 years, older than 70 years, and older than 80 years of age. Furthermore, the extent of comorbidity varied per case, ranging from no comorbidity to serious comorbid conditions. Also the social setting differed in the various cases, from living at home without additional care to living in a nursing home. More detailed information about the case vignettes is provided in Appendix I. The respondents were asked in each case if they would treat the patient with curative intent by means of full-dose chemotherapy or chemotherapy with dose reduction or if they would not treat the patient with curative intent.

In addition, the respondents’ age and gender were assessed as well as the type of hospital they work in. In the Netherlands, three types of hospitals can be discriminated: university hospitals, tertiary medical teaching hospitals, and general hospitals. Tertiary medical teaching hospitals are large teaching hospitals, where highly specialized care is provided [15]. In the Netherlands, 41 % of hematologists work in university hospitals, 32 % in tertiary medical teaching hospitals, 22 % in general hospitals, and in 5 % of hematologists it is unknown.

## Results

### Respondents’ characteristics

Invitations to complete the questionnaire were sent to 255 hematologists. Eighty-six respondents participated in the second part of the survey (33.7 % response rate). Eighty-three respondents fully completed the second part of the survey, and three incomplete questionnaires were returned.

The mean age of the respondents at time of survey was 49.6 years (Table 1). There were more male than female respondents. Of the respondents, 26.7 % worked in a university hospital, 36 % in a tertiary medical teaching hospital, and 37.2 % in a general hospital.

**Table 1:** Sociodemographic characteristics of questionnaire respondents

	Respondents
	*N* = 86
	*N* (%)
Age at time of survey (mean ± SD) (*N* = 60)	49.6 (9.0)
Gender	
Male	56 (65.1)
Female	30 (34.9)
Type of hospital	
University hospital	23 (26.7)
Tertiary medical teaching hospital	31 (36.0)
General hospital	32 (37.2)

### Case vignettes: treatment according to age and comorbidity of patients

**Fig. 1:**
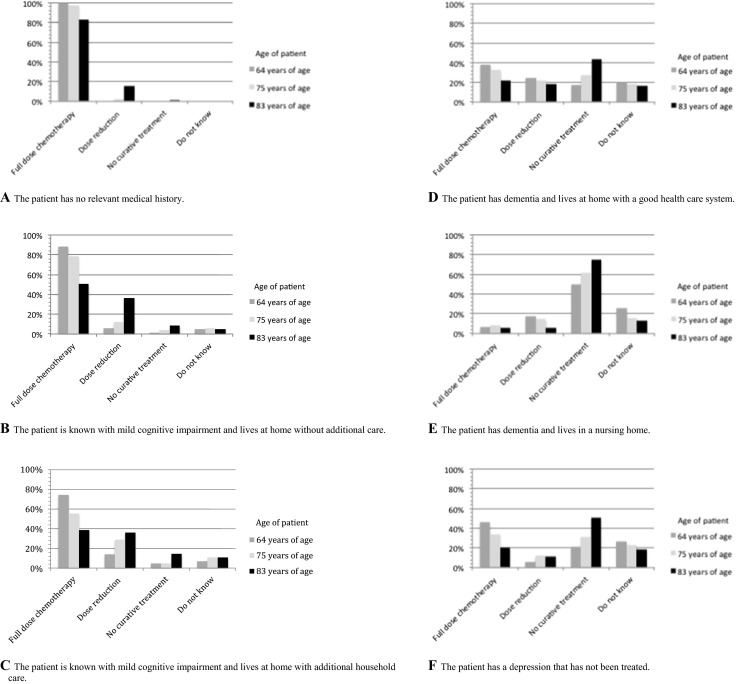

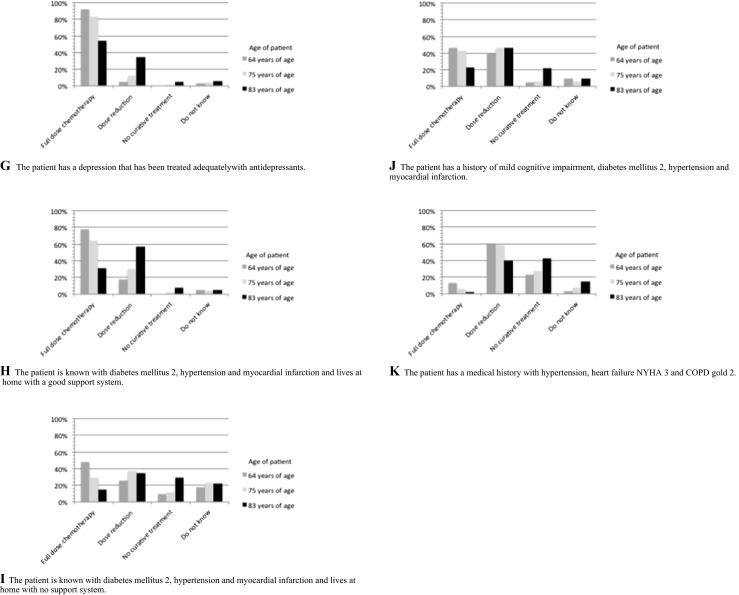
Case vignettes. **a** The patient has no relevant medical history. **b** The patient is known with mild cognitive impairment and lives at home without additional care. **c** The patient is known with mild cognitive impairment and lives at home with additional household care. **d** The patient has dementia and lives at home with a good health care system. **e** The patient has dementia and lives in a nursing home. **f** The patient has a depression that has not been treated. **g** The patient has a depression that has been treated adequately with antidepressants. **h** The patient is known with diabetes mellitus 2, hypertension, and myocardial infarction and lives at home with a good support system. **i** The patient is known with diabetes mellitus 2, hypertension, and myocardial infarction and lives at home with no support system. **j** The patient has a history of mild cognitive impairment, diabetes mellitus 2, hypertension, and myocardial infarction. **k** The patient has a medical history with hypertension, heart failure NYHA 3, and COPD gold 2

The vast majority of respondents would treat an elderly patient diagnosed with DLBCL without a relevant medical history with full-dose chemotherapy irrespective of age (Fig. 1a). However, the percentage decreased from 100 % in “younger” patients to 83 % in patients over 80 years old.

In the presence of mild cognitive impairment, the percentage of patients that would be treated with full-dose chemotherapy decreased in all three age categories, although most patients would still be offered treatment with curative intent (Fig. 1b, c). However, the decrease in treatment with curative intent was more pronounced in elderly patients compared to younger patients.

The majority of respondents would not treat a patient suffering from dementia with full-dose chemotherapy, regardless of age category (Fig. 1d, e). However, younger patients with dementia living at home with a good support system would generally receive chemotherapy with curative intent, while this was not the case in the oldest group of patients (Fig. 1d). Regardless of age, a patient suffering from dementia and living in a nursing home would generally not receive treatment with curative intent (Fig. 1e).

Respondents would not start full-dose chemotherapy in patients with an untreated depression in the majority of cases irrespective of age category (Fig. 1f). In general, if a depression was treated adequately, treatment was not precluded (Fig. 1g).

Patients with a history of diabetes mellitus type 2, hypertension, and myocardial infarction would be treated with curative intent by most of the respondents. However, the number of patients treated with full-dose chemotherapy declined if there was no social support system or concomitant mild cognitive impairment (Fig. 1h–j). In the event of significant comorbidity, most patients would be treated with curative intent by reduced dose chemotherapy, except for the oldest patient category, which frequently would not be treated with curative intent (Fig. 1k).

### Case vignettes: treatment according to type of hospital

**Fig. 2:**
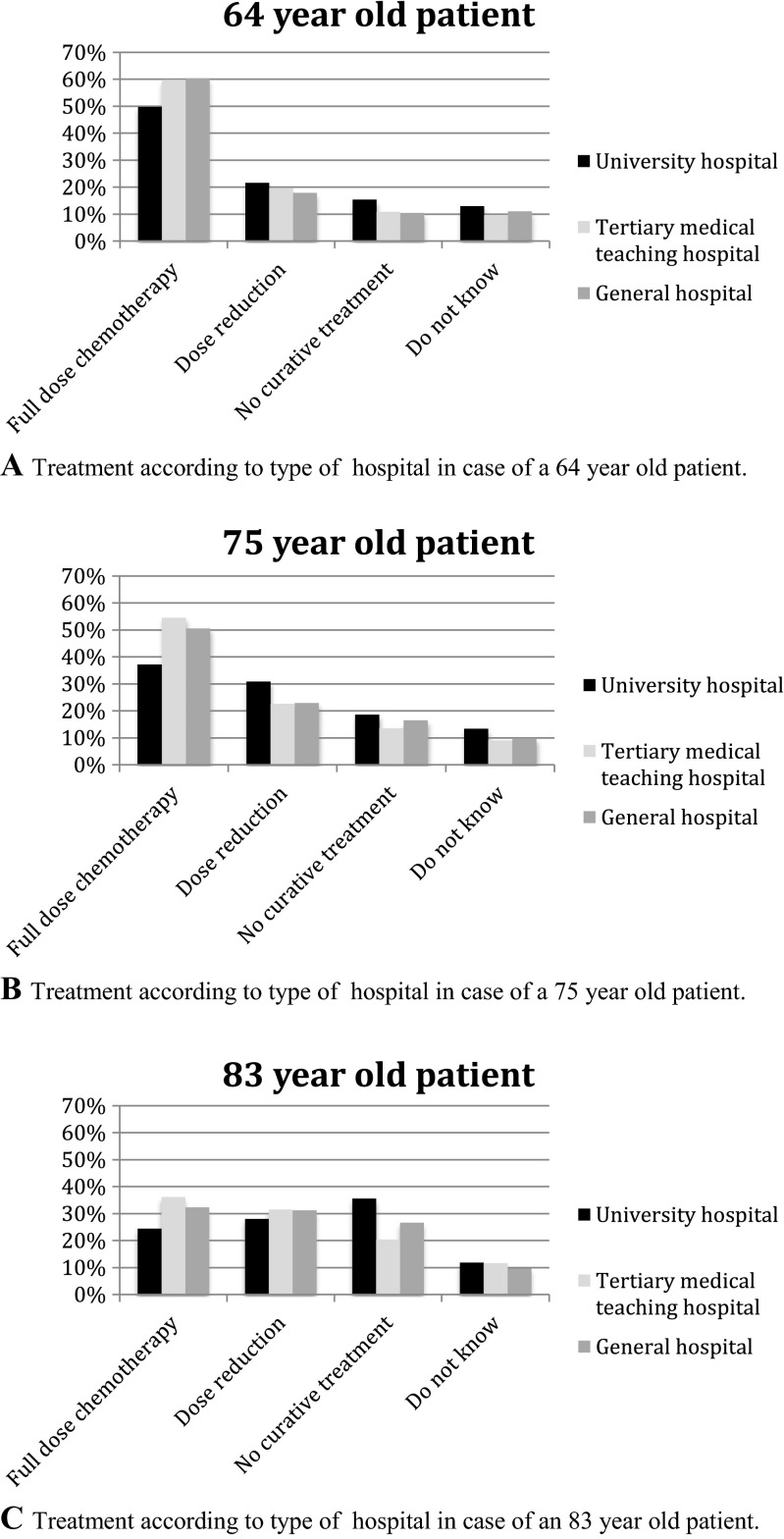
Treatment according to type of hospital and to age of patient irrespective of comorbidity. **a** Treatment according to type of hospital in the case of a 64-year-old patient. **b** Treatment according to type of hospital in the case of a 75-year-old patient. **c** Treatment according to type of hospital in the case of an 83-year-old patient

Respondents working in a university hospital more frequently refrained from full-dose chemotherapy with curative intent in patients of all age categories compared to respondents working in tertiary medical teaching hospitals or general hospitals (Fig. 2a–c). Hematologists in university hospitals more frequently applied dose reductions when treating with curative intent, and in the eldest patient category, they more frequently did not start treatment with curative intent. No major differences were found between respondents working in tertiary medical teaching hospitals and general hospitals.

## Discussion

The aim of the present study was to gain more insight into treatment decision-making by hematologists in DLBCL patients of varying age, comorbidity, and social support by use of case vignettes.

We observed that almost all respondents would treat DLBCL patients without a relevant medical history with curative intent. In the eldest patient category, intentional dose reductions are frequent. This is in line with the results of previous reports [11, 13]. In a recent study among DLBCL patients older than 75 years, dose reductions occurred in 31 % of patients at start of treatment with R-CHOP and age was the most important reason in 27 % of cases [4]. However, in 68 % of patients, there was no clear argumentation.

Furthermore, we found that treatment decision-making is to a large extent influenced by the presence of comorbidity. In case of serious comorbidity, respondents frequently applied dose reductions in advance or refrained from treatment with curative intent. This is in line with the results of the first part of this survey in the same respondents and was also observed is previous studies [5, 9, 13, 16, 17]. In elderly patients, comorbidity is common and a prevalence of up to 87 % in patients aged older than 80 years is described [10]. Comorbidity is associated with lower survival in elderly NHL patients [9, 11, 16–21]. The impaired outcome in patients with comorbidity can not only be the result of the direct impact of comorbidity on outcome but can also be the consequence of less intensive treatment schedules or less treatment tolerability [16, 22].

In addition, the results of this study showed that cognitive impairment has an important influence on treatment decisions. In case of mild cognitive impairment, most patients would be treated with curative intent; however, there was a marked decrease in this percentage in case of dementia. This was most pronounced for the eldest patients above 80 years of age. It has been shown that dementia is associated with an increased mortality rate in NHL patients [9, 23].

Lastly, we observed that depression, especially when not treated adequately, appeared to affect the treatment regime of DLBCL patients. The prevalence of depression in DLBCL patients is high [24]. Moreover, a study among cancer survivors observed increased all-cause mortality in patients with depressive symptoms even after adjustment for major clinical predictors [25]. This might be explained by lower treatment compliance in depressed patients or by a higher incidence of depression in patients with poor performance status [26]. Expected low treatment adherence or worse coping strategies may be reasons for clinicians to treat patients with a depression with adapted chemotherapy schedules.

Interestingly, respondents working in university hospitals seem to treat elderly patients less often with full-dose chemotherapy. This might be the consequence of a referral bias, and possibly these respondents have less experience in treating elderly DLBCL patients.

Respondents declared that comorbidity and cognitive impairment in DLBCL patients largely influence treatment decision-making. In daily clinical practice, the extent of comorbidity and cognitive impairment in a patient is in general judged by the physician without performing a systematic assessment, among others because the latter is time-consuming. Clinical judgment by a physician is however less reliable in detecting geriatric problems compared to a systematic evaluation by comprehensive geriatric assessment (CGA) [27–31]. However, no large prospective randomized controlled trials have been performed investigating the role of CGA in the treatment of elderly DLBCL patients, and therefore, it is not clear how the results of CGA might influence treatment decision-making.

The strengths of our study are that more information is provided about factors that influence treatment decision-making by clinicians, an important topic in cancer treatment. Furthermore, by presenting case vignettes, various situations that resemble daily clinical practice could be studied. Moreover, hematologists of university, tertiary medical teaching hospitals, and general hospitals participated in the study, making the results generalizable. Possible limitations of our study might be that, even though the response rate was reasonable, especially hematologists with a particular interest in this subject responded. In addition, it cannot be entirely excluded that in clinical practice other treatment decisions are made than that the decisions that were indicated in the cases by respondents. At last, an initial treatment decision is not fixed and it is possible that treatment is for example intensified if treatment tolerability appears to be good.

In conclusion, patients without a relevant medical history are in general treated with curative intent irrespective of age. However, in the presence of mild cognitive impairment, dementia, comorbidity, or depression dose reductions in advance are frequently applied or patients are not treated with curative intent. This is most prominent in the eldest patient category.

## Appendix I

**Table 2:** Survey “Treatment of the elderly with a haematologic malignancy”

	Full-dose chemotherapy	Chemotherapy with dose reduction	No treatment with curative intent	Do not know
The patient has no relevant medical history				
The patient is known with mild cognitive impairment and lives at home without additional care				
The patient is known with mild cognitive impairment and lives at home with additional household care				
The patient has dementia and lives at home with a good health care system				
The patient has dementia and lives in a nursing home				
The patient has a depression that has not been treated				
The patient has a depression that has been adequately treated with antidepressants				
The patient is known with diabetes mellitus 2, hypertension and myocardial infarction and lives at home with a good support system				
The patient is known with diabetes mellitus 2, hypertension, myocardial infarction and lives at home with no support system				
The patient has a history of mild cognitive impairment, diabetes mellitus 2, hypertension and myocardial infarction				
The patients has a medical history with hypertension, heart failure NYHA 3 and COPD gold 2				

In the outpatient clinic, you see a 64-year-old patient who is diagnosed with diffuse large B-cell lymphoma. Could you indicate in each of the following cases whether you would treat the patient with curative intent by means of full-dose chemotherapy or chemotherapy with dose reduction or if you would not treat the patient with curative intent? You can also declare that you do not know. The same cases were presented to respondents for a 75-year-old and an 83-year-old patient
